# Supplementary figures and images for: Diquafosol Improves Corneal Wound Healing by Inducing NGF Expression in an Experimental Dry Eye Model
Source: Cells. 2024 Jul 25;13(15):1251. doi: 10.3390/cells13151251 (PMC11311477; doi:10.3390/cells13151251)

Figure 5A

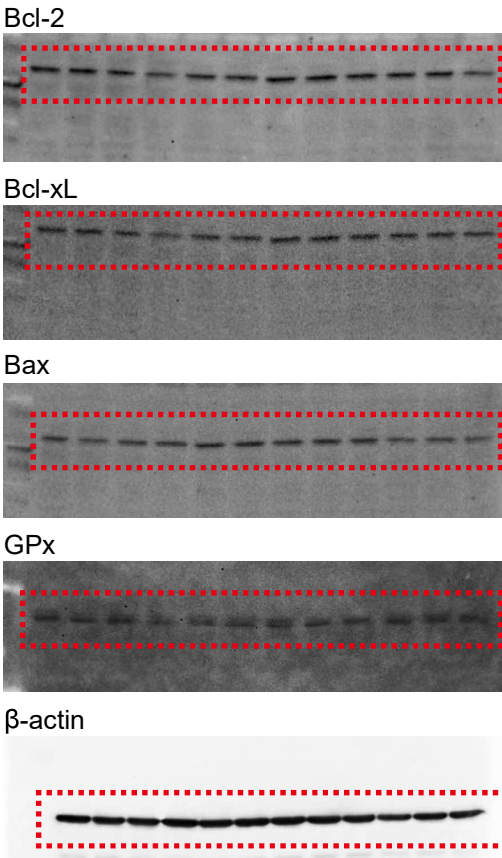

Figure 6A

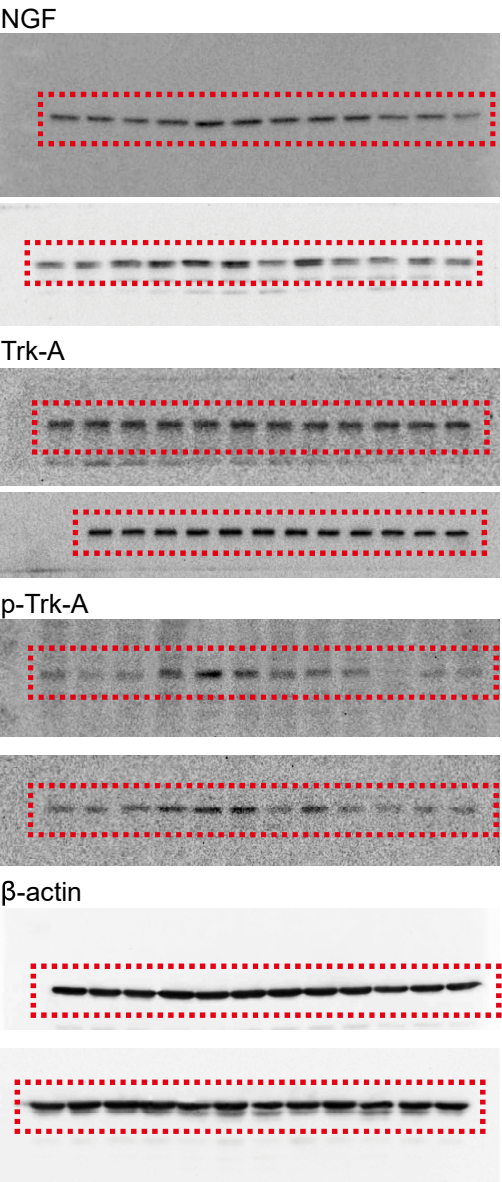

Supplimentary Figure 1

Supplement: Supplementary file 1 [file cells-13-01251-s001.zip › cells-3103926-supplementary.pdf]
